# Modifier genes in *SCN1A*‐related epilepsy syndromes

**DOI:** 10.1002/mgg3.1103

**Published:** 2020-02-07

**Authors:** Iris M. de Lange, Flip Mulder, Ruben van 't Slot, Anja C. M. Sonsma, Marjan J. A. van Kempen, Isaac J. Nijman, Robert F. Ernst, Nine V. A. M. Knoers, Eva H. Brilstra, Bobby P. C. Koeleman

**Affiliations:** ^1^ Department of Genetics Center for Molecular Medicine University Medical Center Utrecht Utrecht The Netherlands; ^2^ Department of Genetics University Medical Center Groningen Groningen The Netherlands

**Keywords:** Dravet, epilepsy, GEFS+, modifier genes, phenotypic variability, *SCN1A*

## Abstract

**Background:**

*SCN1A* is one of the most important epilepsy‐related genes, with pathogenic variants leading to a range of phenotypes with varying disease severity. Different modifying factors have been hypothesized to influence *SCN1A*‐related phenotypes. We investigate the presence of rare and more common variants in epilepsy‐related genes as potential modifiers of *SCN1A*‐related disease severity.

**Methods:**

87 patients with *SCN1A*‐related epilepsy were investigated. Whole‐exome sequencing was performed by the Beijing Genomics Institute (BGI). Functional variants in 422 genes associated with epilepsy and/or neuronal excitability were investigated. Differences in proportions of variants between the epilepsy genes and four control gene sets were calculated, and compared to the proportions of variants in the same genes in the ExAC database.

**Results:**

Statistically significant excesses of variants in epilepsy genes were observed in the complete cohort and in the combined group of mildly and severely affected patients, particularly for variants with minor allele frequencies of <0.05. Patients with extreme phenotypes showed much greater excesses of epilepsy gene variants than patients with intermediate phenotypes.

**Conclusion:**

Our results indicate that relatively common variants in epilepsy genes, which would not necessarily be classified as pathogenic, may play a large role in modulating *SCN1A* phenotypes. They may modify the phenotypes of both severely and mildly affected patients. Our results may be a first step toward meaningful testing of modifier gene variants in regular diagnostics for individual patients, to provide a better estimation of disease severity for newly diagnosed patients.

## Graphical abstract

Relatively common variants in epilepsy genes may play a large role in modulating *SCN1A*‐related phenotypes. They may modify the phenotypes of both severely and mildly affected patients.

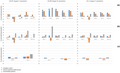

## 1 INTRODUCTION

*SCN1A* (OMIM #182389) is one of the most important epilepsy‐related genes, with pathogenic variants leading to a wide range of phenotypes with varying disease severity (Claes et al., 2003; Escayg & Goldin, 2010; Mulley et al., 2005; Sadleir et al., 2017). One of the most severe associated diseases is Dravet syndrome, which is characterized by intractable epileptic seizures, a diminishing psychomotor development that results in mild to severe intellectual disability (ID), and often walking difficulties and behavioral problems (Brunklaus, Ellis, Reavey, Forbes, & Zuberi, 2012; Dravet, 1978; Rilstone, Coelho, Minassian, & Andrade, 2012). Milder phenotypes include GEFS+ syndrome and febrile seizures, in which seizures show a milder course and a normal intellect is expected (Catterall, Kalume, & Oakley, 2010; Escayg & Goldin, 2010).

*SCN1A* encodes for the *α*‐subunit of a neuronal sodium channel, Nav1.1. Different pathogenic variants in *SCN1A* can have different effects on channel function, which partly explains why the gene is associated with multiple phenotypes. Variants leading to a complete loss of function (LoF) of the channel are virtually always associated with severe phenotypes, whereas milder disturbances in channel function usually cause milder clinical pictures (Meng et al., 2015). However, a large part of the phenotypic variability of patients remains unexplained: there are several reports of families in which multiple members carry the exact same pathogenic *SCN1A* variant, but nevertheless show an intra‐familial variability in phenotype severity (Depienne et al., 2010; Guerrini et al., 2010; Mahoney et al., 2009; Passamonti et al., 2015; Pineda‐Trujillo et al., 2005; Suls et al., 2010). Furthermore, Dravet syndrome patients with similar loss of function variants may show important phenotypic differences, ranging from severely disabled individuals to patients that live much more independent lives (Akiyama, Kobayashi, Yoshinaga, & Ohtsuka, 2010; Harkin et al., 2007; Jansen et al., 2006). This variability makes it difficult to accurately predict clinical outcomes in newly diagnosed young patients, which is very important to parents.

Several factors have been suggested to modify the clinical outcome of *SCN1A*‐related epilepsy and to explain these phenotypic differences. Mosaicism for a pathogenic *SCN1A* variant can have a major ameliorating impact on disease severity (Depienne et al., 2010; Gennaro et al., 2006; de Lange, Koudijs, et al., 2018; Marini, Mei, Helen Cross, & Guerrini, 2006). Furthermore, variants in regulatory regions of *SCN1A* may modulate disease severity (Long et al., 2008; Zeng et al., 2014). Additionally, clinical management and especially the use of contra‐indicated medication can affect clinical outcomes (Ceulemans, 2011; Guerrini et al., 1998; de Lange, Gunning, et al., 2018).

Moreover, variants in modifier genes may influence *SCN1A*‐related phenotypes. An important effect of modifier genes has already been described for several other genetic disorders (Emond et al., 2013; Guo et al., 2015; Vélez et al., 2016), and there are strong indications that genetic background can modulate the clinical effects of pathogenic *SCN1A*‐related phenotypes too, in human patients as well as in *Scn1a* knock‐out mice (Catterall et al., 2010; Depienne et al., 2010; Guerrini et al., 2010; Hawkins & Kearney, 2016; Miller, Hawkins, McCollom, & Kearney, 2014; Pineda‐Trujillo et al., 2005; Scheffer, Zhang, Jansen, & Dibbens, 2009; Singh, Scheffer, Crossland, & Berkovic, 1999; Suls et al., 2010; Yu et al., 2006). Several potential modifier genes have already been identified: variants in *SCN9A, SCN8A, SCN2A, HLF, POLG, KCNQ2, CACNB4, CACNA1G*, and *CACNA1A* might aggravate or partially rescue clinical outcomes (Calhoun, Hawkins, Zachwieja, & Kearney, 2017; Gaily et al., 2013; Hammer et al., 2017; Hawkins & Kearney, 2012, 2016; NA, Martin, Frankel, Kearney, & Escayg, 2011; Martin et al., 2007; Ohmori et al., 2013, 2008; Singh et al., 2009). Potential modifier loci, identified in *Scn1a* knock‐out mice with different disease severities, also contain several candidate modifier genes, including GABA receptor subunit genes, ion channel genes and genes associated with seizures or neuronal hyperexcitability (Miller et al., 2014). Furthermore, an enrichment of rare variants in neuronal excitability genes in general has been identified in severely affected Dravet syndrome patients, compared to mildly affected Dravet syndrome patients (Hammer et al., 2017). However, these potential modifiers each account for only a small portion of the clinical variability of *SCN1A‐*related phenotypes. Many only show an effect when studied in large groups of patients and different patients might be affected by different modifiers or by multiple modifiers simultaneously. Currently, no clinically relevant modifier genes have been identified for which diagnostic testing can be offered, and thus more research is needed to understand clinical variability and to improve the counselling of patients.

Here, we investigate the presence of rare and more common variants in epilepsy‐related genes that could potentially modify disease severity, in a cohort of 87 patients with *SCN1A*‐related epilepsy. We provide a descriptive overview of variants present in patients with phenotypes on the most extreme ends of the spectrum, and furthermore investigate variants in six families with multiple affected members that show varying disease severities.

## 2 MATERIALS AND METHODS

### 2.1 Editorial policies and ethical considerations

The study was approved by the Ethical Committee of the University Medical Center Utrecht. Informed consent was obtained from participants or their legal caretakers, according to the Declaration of Helsinki.

### 2.2 Cohort and clinical data

#### 2.2.1 Participants

A cohort of 87 participants with pathogenic *SCN1A* variants was evaluated, most of whom have been described previously (de Lange, Gunning, et al., 2018; de Lange, Koudijs, et al., 2018). Only participants with pathogenic variants (class V) or likely pathogenic variants (class IV) in *SCN1A* were included, according to the American College of Medical Genetics and Genomics criteria (Richards et al., 2015). All variants had been detected and classified in diagnostic laboratories. Patients that had previously been shown to be mosaic for their pathogenic *SCN1A* variant were excluded (de Lange, Koudijs, et al., 2018).

#### 2.2.2 Disease severity classification

For all participants detailed clinical data were collected from medical records and semi‐structured telephone interviews. Patients were either part of families with multiple *SCN1A* variants carriers, or the only affected member in their family. In all patients absolute disease severity was defined as cognitive functioning at the age of 6 years. We assessed cognitive functioning retrospectively at the age of 6 years old as previously described (de Lange, Gunning, et al., 2018). This was done to limit the influence of an older age on cognitive outcomes, since average cognition declines with age in Dravet syndrome patients. IQ‐ and developmental assessment scores, established at different ages, were interpolated by linear regression, to obtain approximate scores at the age of 6 years. This age was chosen since cognitive decline is generally most severe in the first years following disease onset (Brunklaus et al., 2012; Nabbout et al., 2013). Patients with an IQ or developmental quotient (DQ) of >70 (no or borderline ID) at age 6 were classified as “mild”, while patients with an IQ or DQ of <50 (moderate or severe to profound ID) were classified as “severe”. Patients with an IQ/DQ score of 50–70 were classified as “intermediate”. Participants under the age of 6 years old were not classified, unless they already showed an IQ/DQ of <50. Participants for whom a classification at age 6 could not be reliably obtained were also not classified. Furthermore, if clearly varying phenotypes were present in families with multiple variant carrying family members (different syndromes, or large differences in seizure frequencies or cognitive outcomes), disease severity was defined as “mild” or “severe” relative to other affected family members (e.g., a participant with Dravet syndrome and an unaffected father, both carrying the same pathogenic *SCN1A* variant, would be classified as “relatively severe” and “relatively mild” respectively).

To compare subgroups, we then excluded “mild” patients that did not carry an *SCN1A* variant that was predicted to cause a loss of function (LoF), or a variant that has been described before in Dravet syndrome patients. This limits the influence of the different pathogenic *SCN1A* variants itself on the phenotypes and creates a group of patients for which we can be relatively certain that ameliorating modifiers play a role. The “severe” and “intermediate” groups included patients with all mutation types.

### 2.3 Molecular analyses

#### 2.3.1 Exome sequencing

Whole‐exome sequencing was performed on DNA from lymphocytes in all patients by the Beijing Genomics Institute (BGI), using the Agilent V5 50M exome kit enrichment, followed by paired‐end sequencing on an Illumina Hiseq. The resulting data was processed using an in‐house developed pipeline (Ernst et al., 2017), according to the best practices guidelines (Auwera et al., 2013). Briefly, sequencing reads were mapped using BWA‐MEM v0.7.5a (Li & Durbin, 2009), duplicates were marked and lanes were merged. Next, using GATK IndelRealigner (v3.4–46) (McKenna et al., 2010) indels were realigned and the GATK HaplotypeCaller tool was used to create a GVCF per patient containing SNPs and indels. These GVCFs were jointly genotyped using GATK GenotypeGVCFs for the described cohort. Variants were flagged using GATK VariantFiltration if they did not meet the certain criteria. For SNPs the criteria were: QD <2.0, MQ <40.0, FS >60.0, HaplotypeScore >13.0, MQRankSum <−12.5, ReadPosRankSum <−8.0, snpclusters ≥3 in 35 bp. The criteria for indels were as follows: QD <2.0, FS >200.0, ReadPosRankSum <−20.0. Finally, variants were annotated using SnpSift (v4.3t) and dbNSFP (v3.5).

#### 2.3.2 Filtering of variants

We investigated variants in 422 genes that are all either associated with epilepsy, are implicated to modify epilepsy phenotypes, are associated with neuronal excitability, or function in the same pathway as *SCN1A*, based on epilepsy gene panels used in the University Medical Center Utrecht (EPI00v18.1), previous literature and the KEGG pathway database (https://www.kegg.jp/kegg-bin/show_pathway?ko04728, accessed June 2016) (further referred to as “epilepsy genes”; see Data S1 for the complete list, and Data S2 for characteristics). A distinction was made between established monogenic epilepsy genes (when present in the diagnostic epilepsy gene panel of the University Medical Center Utrecht) (EPI00v18.1) and candidate genes (all other genes). We filtered for PASS‐variants that were predicted to alter protein function (frameshift, stop‐gain, stop‐loss, start‐loss, in‐frame deletion, in‐frame insertion, splice donor, splice acceptor, and nonsynonymous missense variants). Five categories of variants were established (type A–E), based on different minor allele frequencies (MAF) of the variants (in both exomes and genomes in the gnomAD database, r2.0 (Exome Aggregation Consortium et al., 2015), all populations) and on their deleteriousness as predicted by CADD scores (Combined Annotation‐Dependent Depletion, v1.3) (Kircher et al., 2014): Type A variants have a MAF of <0.01 and a (PHRED‐scaled) CADD‐score of >20 (representing the top 1% deleterious substitutions in the human genome); type B variants have a MAF of <0.01 and a CADD‐score of >10 (representing the top 10% deleterious substitutions in the human genome), type C variants have a MAF of <0.01 and any CADD score; type D variants have a MAF of <0.05; and type E variants have a MAF of <0.1. The known pathogenic *SCN1A* variant of each patient was excluded.

The same categories of variants were established for variants in four sets of control genes (control 1: immunodeficiency‐related genes, *n* = 360; control 2: genes related to cardiovascular disease [excluding genes related to conduction abnormalities], *n* = 109; control 3: genes related to kidney disease, *n* = 223; control 4: genes related to either hemostasis, erythroid cell membrane defects, congenital diarrhea, neonatal erythroderma, or angioedema, *n* = 297), and in genes associated with ID (excluding genes also present in the epilepsy gene‐list, *n* = 659), based on genes included in diagnostic gene panels of the University Medical Center Utrecht (version 9, http://www.umcutrecht.nl/NGS) (see Data S1 for the complete lists, and Data S2 for characteristics).

### 2.4 Data analyses

#### 2.4.1 Proportions of variants in epilepsy genes and control genes compared to the ExAC database

We investigated whether groups of patients carry an excess of variants in our selection of epilepsy genes, as compared to the number of variants in the different sets of control genes (1–4). Since these control sets contain different numbers of genes than the set of epilepsy genes, with different lengths and mutation rates, we first investigated a healthy control population to establish the normal ratios of variants between the epilepsy genes and the different control sets. For this, we extracted variants in the same genes from the ExAC database (Exome Aggregation Consortium et al., 2015), using the same filters as applied in our cohort (frameshift, stop‐gain, stop‐loss, start‐loss, in‐frame deletion, in‐frame insertion, splice donor, splice acceptor and non‐synonymous missense variants, with MAFs of either <0.01, <0.05 or <0.1). Only variants present in non‐Finnish Europeans were analyzed, as this population resembles the ethnicity of 97% of our own cohort. Directly comparing numbers of variants found in the ExAC database to other data can lead to incorrect results, as differences in sequencing methods, coverage and variant calling may lead to biases (Barrett et al., 2017). However, we expect the ratios of variants in epilepsy genes and control sets of genes to be roughly similar in both ExAC data and in our own sequencing data, as within each cohort the same protocols are used to analyse the various gene sets. We therefore compare these ratios, rather than absolute numbers of variants, in both cohorts. The ratio of variants in epilepsy genes and in the different sets of control genes in the ExAC database (=numbers of epilepsy gene variants divided by the number of control gene variants) was used to calculate the expected number of variants in epilepsy genes in our cohort (the established ExAC ratio times the number of variants in control genes in our cohort). We compared this expected number of variants in epilepsy genes to the actual number of variants found in our cohort, to obtain the percentage of over‐ or underrepresentation. Fishers' exact test was used to determine whether this over‐ or underrepresentation of epilepsy gene variants was statistically significant (*p*‐value threshold for significance: <.05 divided by the number of tests to the corrected for multiple testing). These analyses were performed for the ratio between epilepsy gene variants and variants in all four sets of control genes, for all three frequency thresholds (<0.01, <0.05 or <0.1; type C, D and E variants) and for different groups of patients (the complete cohort, only patients on the extreme ends of the disease spectrum, and only intermediate patients). We hypothesized that patients with a phenotype on both the severe and mild ends of the disease spectrum would carry more variants in epilepsy genes than intermediate patients, as both groups are likely to have a modified phenotype.

#### 2.4.2 Differences between mild and severe patients

We then assessed the distribution of epilepsy gene variants present in our cohort in the different categories of patients (mild, severe and intermediate). The total number of alleles per group was calculated (=the number of genes in which at least one variant was found in at least one of the patient groups, multiplied by two alleles, multiplied by the number of patients in the group, minus one for each X‐linked gene for each male in the group). The number of found variants per group was then divided by the total number of alleles per group, to obtain a percentage of variants corrected for group size and for male/female ratio (since also variants in X‐linked genes are present). Differences between groups were calculated using Fishers' exact test. Analyses were performed 5 times, once for each category of variants (type A–E). For each of these categories, we furthermore identified in which genes severe patients carried most variants compared to mild patients, and vice versa (Fishers' exact test, based on the numbers of variants and total alleles per gene in each group).

#### 2.4.3 Variants found in families with variable phenotypes and in patients with the most extreme phenotypes

In families with multiple affected family members with different disease severities, we report variants that were present in only severe patients and not in their milder family member(s) (=possible negative modifiers that could aggravate the phenotype) and variants that were present in mild patients but not in their severe family member(s) (=possible positive modifiers that could ameliorate the phenotype). Only the most predicted deleterious variants are described (type A), as it is difficult to prove the influence of more common and milder variants.

We furthermore report the most predicted deleterious variants in the patients with the most extreme phenotypes from the mild and severe groups (IQ at the age of six <30 and all the mild patients [IQ >70]). For each patient, the predicted most deleterious variant in an established epilepsy gene and the predicted most deleterious variant in a candidate gene is reported, based on the highest CADD score. When the variant with the highest CADD score was present in a recessive gene, the highest CADD score in a dominant gene was reported, if these were present.

## 3 RESULTS

For 87 participants whole‐exome data were obtained (see Table S1 for information on *SCN1A* pathogenic variants and clinical data). Coverage values of the analyzed gene sets differed between cohorts (ExAC and the described cohort), but was similar for sets within the same cohort (Data S3 and S4). In all patients, their known *SCN1A* pathogenic variants could be identified, except for large structural variants (e.g., deletions of the complete *SCN1A* gene), meaning no samples swaps had occurred.

Varying phenotypes were observed in six families (Data S5). For 69 participants an estimation of cognitive functioning at the age of 6 years old could be made: 22 participants were severely affected, 29 were mildly affected, and 18 patients were categorized as intermediate. For 18 patients no estimation could be made, because they were either under the age of 6 and still mildly affected, or they were severely affected but no official IQ/DQ assessments were available close to the age of 6, meaning we cannot be sure when the exact decline happened. Ten of the mildly affected patients carried a LoF variant or a variant that was previously associated with a severe phenotype, and were included in the “mild” group. The mild group included both brothers from family 3; although one brother is significantly more severely affected than the other, both brothers were still categorized as mild at the age of 6 years old. The “mild” and “severe” patients combined are referred to as “extreme” patients.

### 3.1 Proportion of variants in epilepsy genes and control genes as compared to the ExAC database

**Figure 1:**
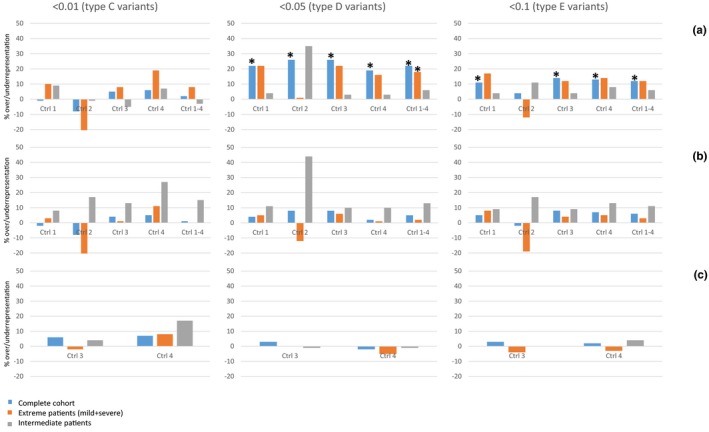
Overrepresentation of variants in epilepsy genes in the cohort. Bars represent the percentage of over‐ or underrepresentation of variants in the different patient groups, based on the ratio of variants found in epilepsy genes and different control groups (ctrl 1, 2, 3, 4 and 1–4), compared to ratios in the ExAC database. (a) Variants in epilepsy genes compared to different control groups; (b) variants in intellectual disability genes compared to different control groups; (c) variants in control group 1 genes compared to control group 3 and 4 (negative control). Results are presented for categories of variants with different allele frequency cut‐offs (<0.01, <0.05, <0.1). Significant values are depicted by asterisks

Table 1 depicts the numbers of variants found in epilepsy genes, ID genes and different sets of control genes, for each groups of patients, in this cohort and in the ExAC database. We observed a significant excess of variants in epilepsy genes in the complete cohort, most strongly for type D variants but also for type E variants, when compared to ratios of variants in the ExAC database (111%–126%, *p* < .0003). A statistically significant overrepresentation of type D epilepsy gene variants was furthermore observed for extreme patients in relation to the control gene sets combined (118%, *p* < .0003). Overall, extreme patients showed a two‐ to fourfold (type E) and five‐ to sevenfold (type D) greater excess of epilepsy gene variants than intermediate patients (Table 2; Figure 1a). This pattern was observed in relation to all sets of control genes except for set 2. No significant excess of variants in intellectual disability genes was observed (Table 2; Figure 1b). There was no significant excess of variants in control set 1 in relation to variants in control set 3 and 4, conform expectation and suggesting validity of these analyses (Table 2; Figure 1c).

**Table 1:** Number of variants found in the ExAC database and in the current cohort for different groups of patients and different categories of variants

Gene set	Type C variants (MAF^a^ <0.01)				Type D variants (MAF <0.05)				Type E variants (MAF <0.1)			
	ExAC	Complete cohort	Extreme patients	Intermediate patients	ExAC	Complete cohort	Extreme patients	Intermediate patients	ExAC	Complete cohort	Extreme patients	Intermediate patients
Epilepsy genes	300,155	851	337	161	556,293	1,876	662	335	805,686	2,455	888	481
Control 1	299,750	858	305	176	641,440	1,778	626	371	950,857	2,608	893	546
Control 2	90,285	278	130	49	185,549	497	218	83	260,406	765	327	140
Control 3	260,659	707	270	147	513,655	1,378	501	300	812,452	2,165	798	467
Control 4	261,763	702	247	131	565,998	1,603	579	331	853,276	2,291	824	473
Control 1–4 (total)	912,457	2,545	952	503	1,906,642	5,256	1,924	1,085	2,876,991	7,829	2,842	1,626
ID genes	557,769	1,572	582	355	1,099,721	3,172	1,132	708	1,707,615	4,919	1,739	1,071

a Minor allele frequency; only variants with a frequency below this threshold in both the exomes and genomes in the gnomAD database are included.

**Table 2:** Overrepresentation of variants in epilepsy genes in the cohort, calculated based on different sets of control genes

Assessed group of genes	Based on control set	Over‐ or underrepresentation of epilepsy gene variants in cohort (% more or less than expected^a^ [*p*‐values^b^])								
		Type C variants (MAF^c^ <0.01)			Type D variants (MAF <0.05)			Type E variants (MAF <0.1)		
		Complete cohort	Extreme patients	Intermediate patients	Complete cohort	Extreme patients	Intermediate patients	Complete cohort	Extreme patients	Intermediate patients
Epilepsy genes	1	−1 (.846)	10 (.221)	9 (.414)	**22 (<.0003)**	22 (<.0005)	4 (.598)	**11 (<.0003)**	17 (.001)	4 (.552)
	2	−8 (.229)	−22 (.017)	−1 (.941)	**26 (<.0003)**	1 (.904)	35 (.016)	4 (.388)	−12 (.044)	11 (.283)
	3	5 (.387)	8 (.329)	−5 (.690)	**26 (<.0003)**	22 (.001)	3 (.721)	**14 (<.0003)**	12 (.019)	4 (.581)
	4	6 (.285)	19 (.038)	7 (.597)	**19 (<.0003)**	16 (.008)	3 (.727)	**13 (<.0003)**	14 (.007)	8 (.257)
	1–4 (total)	2 (.677)	8 (.245)	−3 (.785)	**22 (<.0003)**	**18 (<.0003)**	6 (.374)	**12 (<.0003)**	12 (.005)	6 (.292)
ID genes	1	−2 (.718)	3 (.750)	8 (.042)	4 (.184)	5 (.288)	11 (.101)	5 (.045)	8 (.051)	9 (.097)
	2	−8 (.178)	−28 (.001)	17 (.315)	8 (.127)	−12 (.073)	44 (.002)	−2 (.612)	−19 (.001)	17 (.092)
	3	4 (.405)	1 (.940)	13 (.231)	8 (.025)	6 (.326)	10 (.166)	8 (.003)	4 (.407)	9 (.120)
	4	5 (.280)	11 (.193)	27 (.020)	2 (.561)	1 (.918)	10 (.159)	7 (.006)	5 (.216)	13 (.025)
	1–4 (total)	1 (.784)	0 (1.000)	15 (.041)	5 (.045)	2 (.599)	13 (.011)	6 (.002)	3 (.320)	11 (.008)
Control set genes	3	6 (.298)	−2 (.835)	4 (.738)	3 (.370)	0 (1.000)	−1 (.908)	3 (.323)	−4 (.367)	0 (1.000)
	4	7 (.204)	8 (.393)	17 (.170)	−2 (.535)	−5 (.419)	−1 (.910)	2 (.465)	−3 (.578)	4 (.594)

a Numbers represent the percentage of over‐ or underrepresentation of variants in epilepsy genes, based on a comparison of ratios of variants found in epilepsy genes and in different control groups, in the ExAC database and the current cohort (e.g. the first “7” means that 107% of the expected number of epilepsy genes was identified in our cohort, based on the ratio of variants present in epilepsy genes and control 1‐genes in the ExAC database, and the number of variants in control‐1 genes present in our cohort).

b *p*‐values are based on Fishers' exact tests on the ratios of variants found in the ExAC database and the current cohort. Significant values (*p* < .0003968) are bolded.

c Minor allele frequency; only variants with a frequency below this threshold in both the exomes and genomes in the gnomAD database are included.

**Table 3:** Distribution of variants in the epilepsy genes between groups of patients, for different categories of genes

	Group of patients	Type A variants (CADD^a^ >20/ MAF^b^ <0.01)	Type B variants (CADD >10/MAF <0.01)	Type C variants (all CADD/MAF <0.01)	Type D variants (all CADD/MAF <0.05)	Type E variants (all CADD/MAF <0.10)
% of variant alleles (based on total number of alleles per group)	Mild (*n* = 10)	1.79	2.33	2.76	4.38	5.70
	Severe (*n* = 22)	2.04	2.33	2.76	4.52	5.81
	Mild + severe (*n* = 32)	1.96	2.33	2.76	4.47	5.78
	Intermediate (*n* = 18)	1.95	2.06	2.35	4.03	5.57
*p*‐values Fishers' exact test	Mild versus severe	.456	1	.997	.73	.812
	Mild versus intermediate	.663	.379	.195	.334	.765
	Severe versus intermediate	.783	.294	.112	.109	.491
	Mild + severe versus intermediate	.998	.259	.089	.113	.727

a PHRED‐scaled CADD (combined annotation dependent depletion). A score of >20 represents the top 1% deleterious substitutions in the human genome.

b Minor allele frequency; only variants with a frequency below this threshold in both the exomes and genomes in the gnomAD database are included.

### 3.2 Differences between mild and severe patients

**Figure 2:**
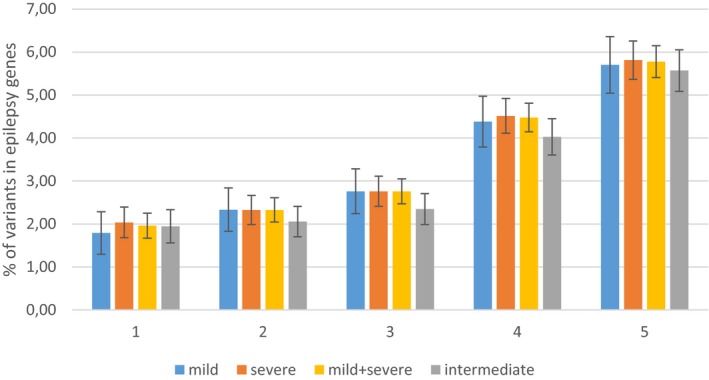
Distribution of variants in epilepsy genes among different groups of patients. Distribution of variants in epilepsy genes among different groups of patients, depicted for different types of variants (A–E). 1: (type A variants) CADD >20/MAF <0.01; 2: (type B variants) CADD >10/MAF <0.01; 3: (type C variants) all CADD/MAF <0.01; 4: (type D variants) all CADD/MAF <0.05; 5: (type E variants) all CADD/MAF <0.10. Percentages of variants relative to the total number of alleles per group are shown

When assessing the distribution of variants present in epilepsy genes in our cohort between the different groups of patients (mild, severe and intermediate), no statistically significant differences were observed (Table 3; Figure 2). This is likely due to smaller sample sizes. We performed a power calculation for type D variants of intermediate patients versus mild and severe patients (the category in which the largest differences were observed), to estimate the effect size that could be reliably detected in a cohort of this size. This analysis showed that, in order to reach statistical significance with a power of 0.8 and our sample size of 50 patients, the group of mild and severe patients combined would have to contain more than six times as much variant alleles as the group of intermediate patients (25% vs. 4.03%). Since only a 1.1 times more variant alleles were observed in the group of mild and severe patients (4.47% vs. 4.03%), our study is likely underpowered to assess the distribution of variants among groups of patients. For each category of variants, we report the genes in which severe patients carried most variants compared to mild patients, and vice versa in Table 4.

**Table 4:** Top 5 genes with an overrepresentation of variants in mild or severe patients, per category of variants

	Type A variants (CADD^a^ >20/ MAF^b^ <0.01)	Type B variants (CADD >10/MAF <0.01)	Type C variants (all CADD/MAF <0.01)	Type D variants (all CADD/MAF <0.05)	Type E variants (all CADD/MAF <0.10)
Genes with an excess of variants in mild patients (gene name [*p*‐value])^c^	*SCN10A* (.027)	*SLC6A8* (.013)	*EFHC1* (.002)	MOCS2 (.003)	MOCS2 (.003)
	*ACTL6B* (.094)	*SCN10A* (.03)	*SCN10A* (.01)	KCNH1 (.008)	KCNH1 (.008)
	*COL3A1* (.094)	*RAI1* (.087)	*SLC6A8* (.013)	EFHC1 (.01)	DRD4 (.009)
	*DEPDC5* (.094)	*ACTL6B* (.094)	*DSC2* (.027)	SLC6A8 (.013)	SLC6A8 (.013)
	*KPNA7* (.094)	*COL3A1* (.094)	*RAI1* (.087)	MYT1 (.027)	CTSD (.026)
Genes with an excess of variants in severe patients (gene name [*p*‐value])	*GPR98* (.049)	*GPR98* (.201)	*GPR98* (.193)	*RYR2* (.025)	*RYR2* (.013)
	*RYR2* (.419)	*CUX1* (.314)	*ANKRD11* (.3)	*CUX1* (.049)	*CUX1* (.049)
	*ITPR1* (.564)	*RYR2* (.417)	*ANK2* (.314)	*KCNB1* (.088)	*KCNB1* (.088)
		*SIK1* (.564)	*CUX1* (.314)	*ANK2* (.094)	*AKAP9* (.094)
		*TSC1* (.564)	*RYR2* (.417)	*AKAP9* (.094)	*ANK2* (.094)

a PHRED‐scaled CADD (combined annotation dependent depletion). A score of >20 represents the top 1% deleterious substitutions in the human genome.

b Minor allele frequency; only variants with a frequency below this threshold in both the exomes and genomes in the gnomAD database are included.

c *p*‐values are based on Fishers' exact test.

### 3.3 Variants found in families with variable phenotypes

Detailed clinical data on the phenotypes of participants from the six families with varying phenotypes are described in Data S4. *Family 1* consists of a severely affected 10 year old proband with Dravet syndrome, and a father with mild epilepsy and normal cognitive functioning. *Family 2* is a large GEFS+ family in which exome sequencing was performed in two mildly affected family members (only febrile seizures/no seizures at all) and in one severely affected family member (severe epilepsy and a severe social‐emotional delay, classified as Dravet syndrome). *Family 3* consists of two brothers with Dravet syndrome, one of whom is more severely affected than the other. *Family 4* consists of a proband with a phenotype on the border of Dravet syndrome and GEFS+, with regression over the years. His father has never had any seizures. *Family 5* consists of two brothers of whom the oldest has severe Dravet syndrome and the youngest has a much milder phenotype. *Family 6* consists of a proband with mild Dravet syndrome, a father with a milder epilepsy‐ and better cognitive functioning but with many social‐ and behavioral problems, and a grandmother who only experienced three seizures in her life.

Type A variants that were only present in either the mild or the severe family members of each family are depicted in Table 5.

**Table 5:** Rare and predicted deleterious variants present in only relatively mildly or severely affected members of families with varying *SCN1A*‐related phenotyes

	Family^a^	Gene	Established epilepsy gene^b^	Variant	MAF^c^	CADD‐phred score^d^
Relatively severely affected family members	1	*PRKACA*		c.452T > C|p.Ile151Thr (missense)	0	26.1
	1	*ITPR1*		c.1435G > A|p.Val479Ile (missense)	0.0047	22.2
	2	*GPR98*		c.3151G > T|p.Asp1051Tyr (missense)	0.0021	26
	3	*DRD4*		c.1016G > A|p.Gly339Asp (missense)	0.0015	25
	3	*DEPDC5*	Yes	c.3551T > A|p.Leu1184Gln (missense)	0	32
	3	*DEPDC5*	Yes	c.3434C > T|p.Ser1145Phe (missense)	0.00007785	21.6
	3	*CREB5*		c.685C > A|p.His229Asn (missense)	0.0003	25.5
	3	*DSG2*		c.166G > A|p.Val56Met (missense)	0.0019	27
	4	*ATF2*		c.977C > T|p.Pro326Leu (missense & splice region)	0.0007	23.5
	5	*GNAS*		c.1648G > A|p.Ala550Thr (missense)	0.00002394	24.3
	5	*OGDHL*		c.2201T > C|p.Phe734Ser (missense)	0.0073	32
	6 (proband and father)	*CREB3*		c.359T > C|p.Leu120Pro (missense)	0.0003	23.6
	6 (proband and father)	*SNTA1*		c.566C > T|p.Ser189Leu (missense)	0.0002	23.6
	6 (proband and father)	*GJA9*		c.22G > A|p.Gly8Arg (missense)	0.0017	29.3
	6 (proband and father)	*LGI2*		c.194C > T|p.Ser65Phe (missense)	0.000005	28.4
	6 (only proband)	*TSC2*	Yes	c.275A > T|p.Glu92Val (missense)	0.001	25.9
	6 (only proband)	*RAI1*	Yes	c.725C > T|p.Pro242Leu (missense)	0.003	24.3
	6 (only proband)	*GABRA3*	Yes	c.766C > T|p.Arg256Trp (missense)	0	29.3
Relatively mildly affected family members	1	*KCNB1*	Yes	c.2266A > C|p.Ile756Leu (missense)	0	23.3
	1	*PKP2*		c.76G > A|p.Asp26Asn (missense)	0.0084	33
	1	*GPR98*		c.9650C > T|p.Ala3217Val (missense)	0.0091	23.6
	1	*SHANK3*	Yes	c.1379_1382delGAAT|p.Arg460fs (frameshift)	0.0021	25.4
	1	*SYNGAP1*	Yes	c.3982_3983insCCCCCCCG|p.Arg1328fs (frameshift)	0	34
	1	*DNM3*		c.2171G > A|p.Arg724His (missense)	0.0059	23.3
	1	*GABRA6*		c.805G > A|p.Val269Ile (missens)	0.0025	28
	2 (father and brother of proband)	*RYR2*		c.4451A > G|p.Tyr1484Cys (missense)	0.000008183	25.5
	3	*PRRT2*	Yes	c.647C > A|p.Pro216His (missense)	0.0005	26.2
	3	*CHD5*		c.5074G > T|p.Gly1692Trp (missense)	0.0002	34
	4	*SLC19A3*	Yes	c.388G > A|p.Val130Met (missense)	0.000004062	21
	4	*SZT2*	Yes	c.8384C > G|p.Thr2795Arg (missense)	0.000008129	27.5
	6	*JUP*		c.1165C > T|p.Arg389* (stop)	0.00003253	37
	6	*GOSR2*	Yes	c.509A > G|p.Asn170Ser (missense)	0.0003	23.3
	6	*DOCK3*		c.5446G > A|p.Val1816Met (missense)	0	22.5
	6	*SLC6A1*	Yes	c.1243C > A|p.Leu415Ile (missense)	0.0025	20.1

* indicates a stop mutation, as per the HGVS nomenclature guidelines that Molecular Genetics & Genomic Medicine requires to be used.

a Members from families 1–6, as described in Data S2. The upper part of the table represents the patients who are relatively severely affected, compared to their other family members; the lower part of the table represents the participants who are relatively mildly affected, compared to their other family members.

b Genes were considered established epilepsy genes when present in the diagnostic epilepsy gene panel of the University Medical Center Utrecht.

c Highest frequency of the variant observed in both exomes and genomes in the gnomAD database (Exome Aggregation Consortium et al., 2015).

d Combined annotation dependent depletion (Kircher et al., 2014): numbers represent PHRED‐scaled CADD scores. CADD scores of >20 represent the top 1% deleterious substitutions in the human genome.

### 3.4 Variants found in patients with the most extreme phenotypes

We further investigated type A variants in patients with the most extreme phenotypes in this cohort (IQ at the age of six <30 or >70): this comprised all 10 “mild” patients, and seven of the “severe” patients. For each patient, their most predicted deleterious variant in both an established epilepsy gene and in an epilepsy candidate gene is depicted in Table 6.

**Table 6:** Predicted most deleterious, rare variants in extremely mild and extremely severe patients

	Patient (IQ at the age of 6)^c^	Established epilepsy genes^a^				Candidate modifier genes^a^			
		Gene	Variant	CADD^d^	MAF^e^	Gene	Variant	CADD^d^	MAF^e^
Mild patients^b^	1 (89.1)	*SCN8A*	c.1925C > T| p.Thr642Met	27.5	0	*EFHC1*	c.661C > T| p.Arg221Cys	33	0.0009
	2 (72.8)	*MOCS2*	c.367C > T| p.His123Tyr	31	0.0036	*AIFM3*	c.496C > T| p.Arg166Trp	34	0.0001
	3 (91.4)	*SCN9A*	c.3770A > G| p.Asn1257Ser	23.9	0.0044	*ARNTL*	c.1376C > T| p.Ser459Phe	29.3	0.00004
	Both 4 and 5 (brothers) (76.6 and 98.2)	*ACTL6B*	c.496G > A| p.Val166Met	25.2	0	—	—	—	—
	Both 6 and 7 (twins) (83.7 and 93.3)	*KPNA7*	c.1353T > G| p.Cys451Trp	26.7	0.00003964	*SCN10A*	c.4849G > T| p.Val1617Phe	28.8	0.00007
	8 (100)	*RAI1*	c.5036C > T| p.Ala1679Val	22.2	0.0006	—	—	—	—
	9 (75.7)	*NPRL3*	c.1123C > T| p.Arg375Cys	24.7	0.00003229	*AIFM3*	c.1123C > T| p.Arg375Cys	32	0.0044
	10 (81.9)	*KMT2A*	c.4972C > G| p.Arg1658Gly	29.6	0.0001	*CACNA1H*	c.2470G > T| p.Ala824Ser	22.8	0.00001
Extremely severe patients^f^	1 (18.9)	*KDM5C*	c.203G > A| p.Arg68Gln	34	0.000005615	*PLCB2*	c.2716G > A| p.Glu906Lys	28.8	0.0028
	2 (22.7)	*TSC2*	c.5383C > T| p.Arg1795Cys	31	0.0015	*PHTF1*	c.1779G > T| p.Trp593Cys	34	0
	3 (25.4)	*KCNQ3*	c.1706A > G| p.Asp569Gly	29.4	0.00002031	*BCAN*	c.2117G > T| p.Arg706Leu	32	0.0026
	4 (25.4)	*NBEA*	c.8350G > T| p.Val2784Phe	32	0.0025	*CACNA1H*	c.6048 + 2_6048+5delTAGG	35	0.00003
	5 (26.7)	*BRAT1*	c.2353C > T| p.Arg785Trp	24.4	0.0029	*GJA9*	c.22G > A| p.Gly8Arg	29.3	0.0017
	6 (27.6)	—	—	—	—	*STXBP5L*	c.1135G > A| p.Val379Met	28.6	0.0054
	7 (28.3)	*SLC6A5*	c.1735A > G| p.Met579Val	25.1	0.0000488	*KCNH8*	c.1414A > G| p.Ile472Val	26.8	0.0001

a A distinction was made between established monogenetic epilepsy genes (when present in the diagnostic epilepsy gene panel of the University Medical Center Utrecht) and candidate genes (all other genes in the epilepsy group).

b Patients with an interpolated IQ at the age of six years old >70 (all patients with an *SCN1A* variant that is predicted to cause complete LoF, or a variant that has been previously described in Dravet syndrome patients).

c IQ‐ and developmental assessment scores, conducted at different ages, were interpolated by linear regression, to obtain approximate scores at age 6 years of age as previsously described (de Lange, Gunning, et al., 2018).

d Combined annotation dependent depletion (Kircher et al., 2014): numbers represent PHRED‐scaled CADD scores (a score of >20 represents the top 1% deleterious substitutions in the human genome).

e Highest frequency of the variant observed in both exomes and genomes in the gnomAD database (Exome Aggregation Consortium et al., 2015).

f Patients with an interpolated IQ at the age of six years old <30.

### 3.5 Comparison of current data and previous literature

A list of all previously implicated modifier genes for *SCN1A‐*related epilepsy is shown in Data S6. The number of variants found in these genes in the current cohort is depicted for two different categories of variants: type A, representing the most deleterious variants with large effect size, and type D, as this is the category in which the largest overrepresentation of variants in epilepsy genes was found in patients with extreme phenotypes.

## 4 DISCUSSION

Despite many efforts, we are still not able to fully explain variable phenotypes caused by similar pathogenic *SCN1A* mutations. More insight in modifying factors is essential for understanding genotype–phenotype relations and for accurate counselling of patients. Besides factors such as mosaicism, variants in regulatory regions, and clinical management, variants in modifier genes are suggested to modify phenotypes. We hypothesized that phenotypes of both severely and mildly affected patients are influenced by modifier genes, as both are on the most extreme ends of the disease spectrum. However, different hypotheses are possible as to which kinds of variants can modify these phenotypes: rare variants with large effects, or multiple more common variants with smaller effects. Previously, rare and/or pathogenic variants in genes involved in neuronal excitability and other known epilepsy genes were suggested to be modifiers (Calhoun et al., 2017; Gaily et al., 2013; Hammer et al., 2017; Hawkins & Kearney, 2012, 2016; Hawkins et al., 2011; Martin et al., 2007; Miller et al., 2014; Ohmori et al., 2008, 2013; Singh et al., 2009). Although such variants may have large effects on phenotypes (a second hit in severely affected patients, or a compensating variant in mildly affected patients), they are unlikely to be present in all patients with extreme phenotypes: none of these single modifier genes has been shown to be clinically relevant in a large patient group (Hammer et al., 2017), Another possibility is the presence of (multiple) more common variants in modifier genes, that each have a smaller effect, but may simultaneously tip the balance over to a milder or more severe phenotype. Especially protective variants in mildly affected patients may not be rare, as they are not necessarily subject to negative selection.

To investigate both of the above described mechanisms, we explored in which categories of variants the largest excess was present in patients with extreme phenotypes, using variant data from the ExAC database. Although exact frequencies of variants may differ between the ExAC cohort and our own, we expected the ratios of variants in epilepsy genes versus unrelated genes to remain similar, which was confirmed when assessing the ratios observed in the different control genes (Figure 1c). We observed the largest overrepresentation of epilepsy gene variants in the MAF <0.05 category (type D variants; Figure 1). This indicates that relatively common variants in epilepsy genes, which would not necessarily be classified as pathogenic, may have a large influence on phenotypes. These results are in line with recent findings (Niemi et al., 2018). Although we defined phenotype severity by cognitive capacities, no significant overrepresentation of variants in ID genes was observed, indicating that their role as modifier genes is limited. This implies that much of the cognitive phenotypic variability is driven by differences in seizure susceptibility, which argues for classifying Dravet syndrome as an epileptic encephalopathy: a syndrome in which epileptiform activities contribute to a progressive cognitive dysfunction. However, genes that are associated with both epilepsy and ID were excluded from the ID gene set. We cannot exclude the possibility that variants in these genes are the most important modifiers of cognitive outcomes. These modifying effects may be caused by either changes in seizure susceptibility, or by a direct effect of these gene variants on cognitive functioning. Unfortunately, we did not have data on seizure severity at the age of six years old. Future prospective studies should include such data to further elucidate this relation. Similar outcomes were observed for comparisons to each control set, except for set 2; this may be due to the much smaller number of genes in this control set.

A surplus of variants in epilepsy genes was observed in patients with extreme phenotypes, but not for intermediate patients, indicating that indeed both the phenotypes of severe as well as mild patients may be under the influence of modifier genes. It is worth noting that our main outcome was cognition at the age of six years old, so a rapid or slow decline in cognition in the first years of disease. This measure may not necessarily completely correspond to long term cognitive outcomes.

The findings above suggest that it is difficult to draw conclusions from testing individual patients for variants in modifier genes: it is hard to prove whether a relatively common variant will have a substantial effect, and if so, what this effect will be, since variants are found in both mild and severely affected patients. For rare, pathogenic variants this may be easier. Although no significant excess of these variants was observed in mild or severe patients, they may still be present in several patients: only one extra variant with a large effect size may be necessary to drastically change outcomes, which is difficult to statistically detect. We therefore also report the type A variants (CADD >20, MAF <0.01) that were detected in the most extremely mild and severe patients, and those that were only present in either the mild or the severe members of affected families, in a descriptive way. Studying families in which the same *SCN1A* variant leads to variable phenotypes has several advantages: not only is the primary influence of different *SCN1A* variants themselves removed from analyses, it also means that variants that are shared between both severe and mild family members can be excluded to have significant effects.

Statistically proving the modifying effects of single genes or even specific variants remains difficult; there are only small numbers of patients with extreme phenotypes and *SCN1A* mutations of which the effect can reliably be predicted, which consequently leads to low detection power. Our study may suffer from this. Furthermore, the variety of different possible modifier genes that may act simultaneously makes it difficult to attribute effects to specific variants. In addition, since some genes can carry both LoF and gain of function (GoF) variants, and also variants that cause no relevant effect at all, they may be incorrectly dismissed as modifier genes when variants are present in both severe, mild, and intermediate patients. Functional testing is required to conclusively prove or disprove any modifying effects of single variants, which is not feasible for all variants detected in this study. However, by presenting the most significant genes in each category of variants (Table 4) and variants that are likely to have the largest effects (Tables 5 and 6), we provide data for future reference. Combined with data from future studies similar to ours, trends in the cumulative data may be detected and groups of patients with similar genotype‐phenotype correlations may be assembled for further research.

Despite a lack of statistical significance, some interesting results were observed in our study in relation to previous literature: a predicted deleterious *SCN8A* variant was detected in an extremely mild patient. *SCN8A* has previously been implicated to ameliorate *SCN1A* phenotypes by restoring normal seizure thresholds (Hawkins et al., 2011; Martin et al., 2007). Furthermore, several severe patients carried variants in genes that were previously described to worsen phenotypes *(POLG*, *SCN2A*, *CACNA1A*, and *CACNA1G),* strengthening those associations (Calhoun et al., 2017; Gaily et al., 2013; Hawkins et al., 2011; Ohmori et al., 2013). *GPR98*, a gene implicated in myoclonic epilepsy (Myers et al., 2018), showed the highest overrepresentation of variants in severe patients in three categories of variants, and *SCN10A*, another sodium channel alpha‐subunit gene, was most often implicated in mild patients. One relatively severe patient carried a *GABRA3* variant (family 6); several GABA receptor genes have already been suggested as potential *SCN1A* modifiers (Miller et al., 2014). Inhibition of *DOCK3*, in which a variant was found in a relatively mild patient (family 6), was previously shown to decrease epileptic activity (Li et al., 2016). Interestingly, frameshift variants in *SHANK3* and *SYNGAP1* were detected in a relatively mild patient (family 1). Both genes are associated with severe neurodevelopmental disorders (Carvill et al., 2013; Durand et al., 2007). The presence of these variants in a mildly affected patient may be explained by their location in the genes: the *SHANK3* variant resides in exon 11, which has previously been implicated to be absent from most or all *SHANK3* transcripts (Kolevzon et al., 2011). The *SYNGAP1* variant is at the 3' end of the gene, which may lead to less severe effects. Nevertheless, it remains a possibility that some of the presented variants are sequencing or calling errors, since it was not feasible to confirm all variants by Sanger sequencing. We however do not expect such variants to influence our main results, since similar error rates are to be expected between different groups of patients and categories of variants.

In conclusion, our results indicate that relatively common variants in epilepsy genes, which would not necessarily be classified as pathogenic by themselves, play a large role in modulating phenotypes, in both severely and mildly affected patients. Studies in larger cohorts, combined with functional assessments, will be necessary to confirm or disprove the modifying effects of the genes implicated in this study. Our results may be a first step towards meaningful testing of modifier gene variants in regular diagnostics for individual patients, to provide a better estimation of disease severity for newly diagnosed patients.

## CONFLICTS OF INTEREST

None declared.

## AUTHOR CONTRIBUTION

Conceived and designed the study: EHB, BPCK; Data collection: IML, FM, RS, ACMS, MJAK, IJN, and FRE; Statistic analysis: IML; Wrote the paper: IML. All authors read and approved the final manuscript.

## Ethical Publication Statement

We confirm that we have read the Journal's position on issues involved in ethical publication and affirm that this report is consistent with those guidelines.
