# Supplementary material for: Modifier genes in SCN1A‐related epilepsy syndromes
Source: Mol Genet Genomic Med. 2020 Feb 7;8(4):e1103. doi: 10.1002/mgg3.1103 (PMC7196470; doi:10.1002/mgg3.1103)
Supplement: Supplementary file 1 [file MGG3-8-e1103-s001.docx]

Supplementary Table 1. Characteristics of the study population

|  | Complete cohort | Mildy affected patients | Intermediate patients | Severely affected patients |
| --- | --- | --- | --- | --- |
| n | 87 | 10 | 18 | 22 |
| Age (years, mean, range) | 19 (2-67) | 20 (8-37) | 19 (8-31) | 16 (4-47) |
| Sex: male (n) | 49 | 6 | 10 | 11 |
| *SCN1A* mutation type (n)  -missense  -pore region/loop  -elsewhere  -splicing  -nonsense/frameshift/rearrangement | 31  20  11  9  47 | 2  1  1  0  8 | 4  2  2  0  14 | 7  2  5  2  13 |
| IQ at the age of 6 years old (mean, range) | 58 (19-100) | 84 (73-98) | 57 (50-69) | 34 (19-35) |
| Age at seizure onset (months, mean, range) | 6.7 (1-24) | 7 (4-10) | 5.8 (2-11) | 5 (1-10) |
| Age at first observation of developmental delay (n)  -<12 months  -12-23 months  -24-35 months  -36-47 months  -≥48 months  -no delay  -missing | 11  26  16  12  7  13  2 | 0  1  4  1  3  1  0 | 3  8  2  4  1  0  0 | 6  10  5  0  0  0  1 |
| Age at first afebrile seizure2 (n)  -<12 months  -12-23 months  -24-47 months  -≥48 months  -never  -missing | 42  17  8  9  4  7 | 0  4  1  3  0  2 | 13  1  2  1  0  1 | 15  4  0  1  0  2 |

^1^Absences, myoclonias, focal seizures and focal seizures with impaired awareness.
^2^Defined as seizures with a body temperature below 38°C, or seizures for which medical records reported “no fever” without mentioning the exact temperature.
