# Supplementary material for: Modifier genes in SCN1A‐related epilepsy syndromes
Source: Mol Genet Genomic Med. 2020 Feb 7;8(4):e1103. doi: 10.1002/mgg3.1103 (PMC7196470; doi:10.1002/mgg3.1103)
Supplement: Supplementary file 3 [file MGG3-8-e1103-s003.pdf]

2. Characteristics of epilepsy genes, ID genes and control sets 1-4

Gene set	Average z-score missense	Average pLI LoF	Average number of exons	Average number of coding basepairs	% of genes pLI >= 0.9	Total number of coding basepairs
**Epilepsy genes**	2.305	0.596	16.5	2559	47.5	1080053
**Control 1**	0.891	0.359	13.4	1962	26.4	700293
**Control 2**	1.409	0.469	16.9	2506	36.7	273194
**Control 3**	0.409	0.263	17.3	2679	21.1	597507
**Control 4**	0.635	0.297	14.3	2031	21.1	603202
**ID genes**	1.293	0.395	15.1	2404	32.3	1576863
