# Supplementary material for: Modifier genes in SCN1A‐related epilepsy syndromes
Source: Mol Genet Genomic Med. 2020 Feb 7;8(4):e1103. doi: 10.1002/mgg3.1103 (PMC7196470; doi:10.1002/mgg3.1103)
Supplement: Supplementary file 4 [file MGG3-8-e1103-s004.pdf]

3. Coverage of epilepsy genes, ID genes and control sets 1-4 in the current cohort

Gene set	Average coverage complete cohort	% of basepairs >20X complete cohort	Average coverage extreme patients	% of basepairs >20X extreme patients	Average coverage intermediate patients	% of basepairs >20X intermediate patients
**Epilepsy genes**	98.0	95.6	102.9	95.7	85.7	94.8
**Control 1**	96.3	93.9	101.0	93.9	83.9	93.1
**Control 2**	99.2	97.0	104.1	97.0	86.8	96.4
**Control 3**	96.6	95.2	101.3	95.2	84.6	94.5
**Control 4**	98.6	96.7	103.5	96.8	86.2	96.0
**ID genes**	100.3	95.9	105.3	96.0	87.6	95.2
