# Supplementary material for: Modifier genes in SCN1A‐related epilepsy syndromes
Source: Mol Genet Genomic Med. 2020 Feb 7;8(4):e1103. doi: 10.1002/mgg3.1103 (PMC7196470; doi:10.1002/mgg3.1103)
Supplement: Supplementary file 5 [file MGG3-8-e1103-s005.pdf]

4. Coverage of epilepsy genes, ID genes and control sets 1-4 in the ExAC database

Gene set	Average coverage ExAC	Median coverage ExAC
**Epilepsy genes**	53.2	55.0
**Control 1**	53.4	55.5
**Control 2**	55.5	58.7
**Control 3**	54.8	57.6
**Control 4**	54.9	57.1
**ID genes**	53.5	55.8
