# Supplementary material for: Modifier genes in SCN1A‐related epilepsy syndromes
Source: Mol Genet Genomic Med. 2020 Feb 7;8(4):e1103. doi: 10.1002/mgg3.1103 (PMC7196470; doi:10.1002/mgg3.1103)
Supplement: Supplementary file 6 [file MGG3-8-e1103-s006.pdf]

5. Clinical descriptions of families with varying *SCN1A* phenotypes

Family 1

The proband of family 1, a 10 year old boy, started having febrile seizures at the age of 10 months. Soon after, afebrile seizures started occurring, and his epilepsy became intractable. He currently experiences multiple generalized tonic-clonic-, tonic-, absence seizures and myoclonias per week, while using valproate and clobazam. Status epilepticus has occurred twice. The longest period of seizure freedom since onset was 1.5 weeks. A developmental delay became evident at age 3 years old. At age 6.5 years old his developmental age was estimated to be around 2.5 years. He shows behavioural problems, is diagnosed with autism, and furthermore has walking difficulties caused by ataxia and hypotonia. At age 4 years old a pathogenic *SCN1A* variant was found (c.1186G>T (p.Gly396Trp)) and Dravet syndrome was diagnosed.

The father of this boy, a 43 year old man, was found to carry the same *SCN1A* variant. He has experienced seizures since the age of 18 months, at most 4 per year and in his childhood only occurring during fever. He was treated with valproate, which was discontinued between 12 and 16 years of age. At age 16 seizures reoccurred, after which valproate was prescribed again. With that, seizure freedom was established until the age of 28, after which 6-7 seizures occurred in 6 months. Currently, this patients uses levetiracetam and carbamazepine and has had no seizures for the last 11 years. He has only ever experienced generalized tonic-clonic seizures and there was never a status epilepticus. There has been no developmental delay and this patient has followed regular education.

Family 2

Family 2 is a large GEFS+ family in which multiple family members experienced febrile seizures, mild epilepsy or no symptoms at all, based on a pathogenic *SCN1A* variant (c.1217T>C (p.Val406Ala)). Three family members have been included in this study. Two of those, a father (age 64) and son (age 35), showed phenotypes comparable to most other affected family members. The father has never experienced any seizures, as far as he knows, and had a normal development. His son has experienced a febrile seizure two or three times between the age of 1-2 years, for which he was never treated. He has followed regular education also had a normal development. His sister however was severely affected compared to her other family members. Seizure onset was at age 18 months and tonic-clonic seizures occurred weekly. She soon also developed focal seizures with impaired awareness, at age 8 absences started to occur, and at age 15 she developed myoclonias. A status epilepticus has occurred 16 times. She had been treated with many anti-epileptic drugs, but became seizure free for 6 years using a combination of topiramate, oxcarbazepine and clobazam. Although she scored well on cognitive assessments (IQ 95 at age 29) she followed special education and was impaired on a social-emotional level, for which a developmental age of 2-4 years old was established. She showed behavioural problems and signs of ADHD. Ataxia was present, leading to walking difficulties, and she furthermore suffered from Crohn's disease, psoriasis and asthma. She committed suicide at the age of 30.

Family 3

Family 3 consists of two brothers that both carry the same pathogenic *SCN1A* variant (c.1209delT (p.Phe403fs)). The oldest brother (age 37) had a first febrile seizure at the age of 9 months. Soon after he developed intractable generalized tonic-clonic seizures. During puberty he

developed focal seizures with impaired awareness. He has never experienced myoclonias or a status epilepticus. He currently uses valproate and topriamate and is now free of seizures. There was a slowing of development and he has followed special education, although an IQ of 91 was assessed. He works in a protected environment.

His younger brother (33 years old) is more severely affected. He also experienced a first febrile seizure at the age of 9 months, and afterwards developed intractable epilepsy with mostly tonic-clonic seizures. He has experienced a status epilepticus 6 times and has recently developed focal seizures with impaired awareness, myoclonias and absences. He is diagnosed with an autism spectrum disorder, followed special education and an IQ of 56 was assessed. He attends day-care activities.

Family 4

The proband in family 4 is a 28 year old man that experienced 6 febrile seizures between the ages of 18 months and 5 years. Between the ages of 5 and 16 years tonic-clonic seizures became provoked by exercise, and seizures are currently still occurring 10 times per year. The longest period of seizure freedom was 5 months. He currently uses pregabalin, valproate, carbamazepine, clobazam an topiramate. He experienced status epilepticus as a young child, at 7-8 years old and at 10 years old. Although this patient had a normal development as a child, during puberty he was not able to keep up with classmates anymore and memory problems became an issue. He originally started high school at a high educational level, but had to drop down several levels and now works in a sheltered environment. A pathogenic *SCN1A* variant (c.1719C>A (p.Ser573Arg)) was detected at age 28.

His father (age 67) carries the same variant, has never experienced any seizures and has followed regular education without any signs of developmental delay.

Family 5

Family 5 consists of two brothers that both carry a pathogenic *SCN1A* variant (c.2584C>T (p.Arg862*)). The oldest brother (8 years old) developed intractable seizures at the age of 12 months. He currently experiences tonic-clonic seizures twice per week, and has also developed focal seizures with impaired awareness, absences (twice per day), myoclonias and tonic seizures, while using valproate, clobazam and stiripentol. His longest period of seizure freedom was 3 months. He has experienced convulsive status epilepticus six times, and has had multiple occurrences of non-convulsive status epilepticus per year. His development started slowing at age 18 months, and at 8 years old his developmental age was estimated to be 24 months. No behavioural problems are reported. He has difficulties walking, characterized by ataxia and hypotonia.

His younger brother (age 4) also developed seizures at the age of 12 months, but experiences them much less frequently. He currently has around 6 tonic-clonic seizures and absences per year, while only using valproate. His longest period of seizure freedom was 7 months and he has experienced status epilepticus twice. He develops without any signs of delay and follows regular education. He shows no walking difficulties or behavioural problems.

Family 6

Family 6 consists of three members, all carrying a pathogenic *SCN1A* variant (c.2945T>C (p.Val982Ala)). The proband, a 9-year old boy, started having seizures at the age of 6 months, during a fever episode. He developed multiple seizure types (focal seizures with impaired

awareness, atonic seizures, hemi-convulsions, absences, tonic seizures and myoclonias) but mostly suffered tonic-clonic seizures that could occur multiple times per day. He experienced status epilepticus once and has temporarily lived in an epilepsy center during weekdays. He has now however been seizure free since 2.5 years, while using valproate, topiramate and clobazam. A developmental delay was noticed at the age of 3 years and an IQ of approximately 70 was tested. He follows special education, shows behavioral problems and signs of ADHD. There are no walking disabilities.

His father, a 32 year old man, has a less severe epilepsy phenotype. He experienced febrile seizure in his early youth and afebrile seizures as a child, but has also been seizure free for long periods of time. He only ever experienced tonic-clonic seizures and has never had a status epilepticus. He currently uses valproate and clobazam, but does not take his medication regularly and lives an unhealthy irregular life, which has caused an increase in seizure frequency to once a month. He has followed normal education although some support was necessary, and works as a cook. He is however diagnosed with an autism spectrum disorder, shows signs of ADHD, has problems with social functioning, struggles with addiction, has debts, is homeless and can show aggressive behavior.

His mother, the grandmother of the proband (age 55), has a much milder phenotype than her son and grandson. She has only experienced 3 tonic-clonic seizures in her life, for which she never used any medication, and has been seizure free for 35 years now. She is highly educated and shows no behavioural problems.

The grandmother of the proband has a deceased brother for whom no DNA was available. He had a severe epilepsy phenotype with 12-13 seizures per day and intellectual disability. He drowned during an epileptic seizure. *SCN1A* testing was never performed.
