# Supplementary material for: Modifier genes in SCN1A‐related epilepsy syndromes
Source: Mol Genet Genomic Med. 2020 Feb 7;8(4):e1103. doi: 10.1002/mgg3.1103 (PMC7196470; doi:10.1002/mgg3.1103)
Supplement: Supplementary file 7 [file MGG3-8-e1103-s007.pdf]

6. Overview of previously described candidate modifier genes and variants in them detected in the current cohort

Previously described candidate gene	Described effect	Reference	Number of variants (CADD[a] >20) in current cohort				
			Variants in only mild or severe member of families with varying phenotypes (MAF<0.01, CADD>20)	*Type A variants (MAF[b]<0.01, CADD>20)*		*Type D variants (MAF<0.5, all CADD scores)*	
				Mild patients (n)	Severe patients (n)	Mild patients (n)	Severe patients (n)
**SCN9A**	Pathogenic variants present in multipe Dravet syndrome patients	Singh 2009		1	2	2	3
**SCN8A**	SCN8A pathogenic variants rescue SCN1A-phenotype in mice; increased resistance for induces seizures in mice with GEFS+ variants	Martin, 2007; Hawkins, 2012		1		1	1
**HLF**	Decreased survival in HLF/SCN1A double knockout mice	Hawkins, 2016					
**POLG**	POLG variants may increase susceptibility to focal brain injury during prolonged seizures in Dravet syndrome	Gaily,2013			1		4
**CACNB4**	Pathogenic variant in Dravet syndrome patient who died after status epilepticus	Ohmori, 2008					
**CACNA1G**	Decreased Cacna1g expression led to partial amelioration in SCN1A+/− mice	Calhoun, 2017			1	2	2
**CACNA1A**	More severe phenotype in Dravet syndrome patients who also have CACNA1A variants	Ohmori, 2013			1		3

**GABRA2**	Potential candidate gene at locus linked to premature lethality in Scn1a+/− mice	Miller, 2014					
**GABRG3**	“ “	Miller, 2014				1	
**GABRB3**	“ “	Miller, 2014					
**GABRA6**	“ “	Miller, 2014		1		2	2
**GABRB2**	“ “	Miller, 2014					1
**CACNA1A**	“ “	Miller, 2014					
**CACNA2D1**	“ “	Miller, 2014					1
**CLCN3**	“ “	Miller, 2014					
**KCNJ11**	“ “	Miller, 2014				1	
**ATP1A3**	“ “	Miller, 2014			1		2
**LGI2**	“ “	Miller, 2014					2
**MAPK10**	“ “	Miller, 2014					
**RELN**	“ “	Miller, 2014					3
**SLC7A10**	“ “	Miller, 2014				1	
**KCNQ2**	Variants present in 3/12 severe Dravet syndrome patients, not in mild patients; More severe phenotype in GEFS+ mice that also carry KCNQ2 variant	Hammer, 2017; Hakwins, 2012				1	3
**SCN2A**	More severe phenotype in GEFS+ mice that also carry SCN2A variant	Hawkins, 2012			1		1
**Top ranking EE genes in common epilepsy**							
**DEPDC5**	Enriched in common epilepsies	Epi4K consortium, 2017	2 variants in more severe brother of family 3; both are however mildly affected			4	5
**LGI1**	“ “	Epi4K consortium, 2017					
**PCDH19**	“ “	Epi4K consortium,					

		2017					
**GRIN2A**	“ “	Epi4K consortium, 2017					2
**KCNA2**	“ “	Epi4K consortium, 2017				2	1
**GABRB3**	“ “	Epi4K consortium, 2017					
**GABRA1**	“ “	Epi4K consortium, 2017					
**KCNQ2**	Enriched in common epilepsies	Epi4K consortium, 2017				1	3
**GABRG2**	Enriched in common epilepsies	Epi4K consortium, 2017					
**SCN1B**	“ “	Epi4K consortium, 2017					
**SLC6A1**	“ “	Epi4K consortium, 2017	1 variant in mildly affected grandmother of Dravet syndrome patients (family 6)				
**EEF1A2**	“ “	Epi4K consortium, 2017					

[a] PHRED-scaled CADD (Combined Annotation Dependent Depletion). A score of >20 represents the top 1% deleterious substitutions in the human genome.

[b]Minor allele frequency; only variants with a frequency below this threshold in both the exomes and genomes in the gnomAD database are included.
